# Q-Switch Nd:YAG Laser-Assisted Decontamination of Implant Surface

**DOI:** 10.3390/dj7040099

**Published:** 2019-10-01

**Authors:** Melanie Namour, Marwan El Mobadder, Delphine Magnin, André Peremans, Tim Verspecht, Wim Teughels, Laurent Lamard, Samir Nammour, Eric Rompen

**Affiliations:** 1 Department of Dental Science, Faculty of Medicine, University of Liege, 4000 Liege, Belgium; melanienamour@gmail.com (M.N.); marwan.mobader@gmail.com (M.E.M.); erompen@hotmail.be (E.R.); 2 Bio- and Soft Matter Division, Institute of Condensed and Nanosciences, Université Catholique de Louvain (UCL), 1348-Louvain-la-Neuve, Belgium; delphine.magnin@uclouvain.be; 3 Laboratoire Physique de la Matière et du Rayonnement (P.M.R.), Université de Namur, 5000 Namur, Belgium; Andre.peremans@gmail.com; 4 Department of Oral Health Sciences, University of Leuven (K.U. Leuven), 3000 Leuven, Belgium; tim.verspecht@kuleuven.be; 5 Department of Oral Health Sciences, University of Leuven (K.U. Leuven) and Dentistry, University Hospitals Leuven, 3000 Leuven, Belgium; wim.teughels@kuleuven.be; 6 Laserspec Center, 5000 Namur, Belgium; Lamard@laserspec.be

**Keywords:** biofilm, dental implant, peri-implantitis, titanium surfaces decontamination

## Abstract

Peri-implantitis (PI) is an inflammatory disease of peri-implant tissues, it represents the most frequent complication of dental implants. Evidence revealed that microorganisms play the chief role in causing PI. The purpose of our study is to evaluate the cleaning of contaminated dental implant surfaces by means of the Q-switch Nd:YAG (Neodymium-doped Yttrium Aluminum Garnet) laser and an increase in temperature at lased implant surfaces during the cleaning process. Seventy-eight implants (titanium grade 4) were used (Euroteknika, Sallanches, France). Thirty-six sterile implants and forty-two contaminated implants were collected from failed clinical implants for different reasons, independent from the study. Thirty-six contaminated implants were partially irradiated by Q-switch Nd:YAG laser (1064 nm). Six other contaminated implants were used for temperature rise evaluation. All laser irradiations were calibrated by means of a powermetter in order to evaluate the effective delivered energy. The irradiation conditions delivered per pulse on the target were effectively: energy density per pulse of 0.597 J/cm^2^, pick powers density of 56 mW/cm^2^, 270 mW per pulse with a spot diameter of 2.4 mm, and with repetition rate of 10 Hz for pulse duration of 6 ns. Irradiation was performed during a total time of 2 s in a non-contact mode at a distance of 0.5 mm from implant surfaces. The parameters were chosen according to the results of a theoretical modeling calculation of the Nd:YAG laser fluency on implant surface. Evaluation of contaminants removal showed that the cleaning of the irradiated implant surfaces was statistically similar to those of sterile implants (*p*-value ≤ 0.05). SEM analysis confirmed that our parameters did not alter the lased surfaces. The increase in temperature generated at lased implant surfaces during cleaning was below 1 °C. According to our findings, Q-switch Nd:YAG laser with short pulse duration in nanoseconds is able to significantly clean contaminated implant surfaces. Irradiation parameters used in our study can be considered safe for periodontal tissue.

## 1. Introduction

Peri-implantitis (PI) is an inflammatory disease of peri-implant tissues, it represents the most frequent complication of dental implants [1]. The reported prevalence for periimplantitis ranges between 28% and 56% [2]. Histological specifications around the implant and implant-related factors lead to a more complex physiopathology of the peri-implantitis compared to the inflammation around natural teeth [3]. According to the World Workshop held by the American Academy of Periodontology and the European Federation of Periodontology, Peri-implantitis presents evidence of visual inflammatory changes in the peri-implant soft tissues combined with bleeding on probing and/or suppuration, increasing probing depths compared with the time of placement of the prosthesis and progressive bone loss [4]. It is important to differentiate peri-implantitis from peri-implant mucositis. The World Workshop defines peri-implant mucositis as a disease in which inflammation of the soft tissues surrounding a dental implant is present without additional bone loss after the initial bone remodeling that may occur during healing following the surgical placement of the implant [4].

Evidence revealed that microorganisms are the chief cause of peri-implantitis [5]. In fact, in peri-implantitis, the surface of the implant becomes contaminated with biofilm, calculus, toxins and organic materials, which seems to prevent any future bone integration. Therefore, the cleaning of the contaminated surface of the implants may allow the integration of newborn periodontium [6].

Moreover, the pathogenic bacteria associated with periimplantitis are more complex than those found under healthy peri-implant conditions and more diverse than those of periodontitis [7]. Therefore, the presence of pathogenic microorganisms is fundamental for the development of periimplantitis [8]. In addition, risk factors such as smoking (i.e., the greatest risk factor), genetics (i.e., the familial history of periodontitis), lack of compliance and poor oral hygiene, systematic disease, iatrogenic causes, soft tissue defects or poor-quality soft tissue at the area of implantation and others are associated with the appearance of peri-implantitis [9].

In periodontitis, non-surgical and surgical treatments of periimplantitis are considered accepted strategies since, as the aforementioned, bacterial colonization of implant surfaces plays an important role in periimplantitis pathogenesis [10]. The treatment of peri-implant infections comprises conservative (non-surgical) and surgical approaches. A mechanical implant cleaning with titanium or plastic-curettes, ultrasonic (with or without Teflon tip) or air polishing [11] as well as local antiseptic medication (chlorhexidine, hydrogen peroxide, sodium percarbonate, povidone-iodine) are also used for the treatment of PI.

As an adjunction to the mechanical implant cleaning treatments, innovative techniques are being reported. One of the most reported methods is the use of lasers. The use of lasers is considered effective and suitable for inflammatory and infectious conditions [12] because of its ablative or vaporization effect, microbial destruction, and biological effects, such as photobiomodulation. Consequently, the bacteria are evaporated, destroyed or denatured by laser irradiation, resulting in their devitalization or inactivation [13,14,15]. Antimicrobial photodynamic therapy (PDT) that consists of the application of photosensitizer and low-level laser or light emitting diode (LED) light energy source to destroy pathogenic bacteria by free-radicals release is a promising technique that is being widely used for the management of periodontitis and mucositis [16]. Unfortunately, the PDT allows germs destruction without any implant surface cleaning. On the other hand, Er:YAG (Erbium-doped yttrium aluminium garnet) laser (laser irradiation with several passages showed promising results in the cleaning of implant surfaces [17]. Though, the reduced flexibility of the Er:YAG laser fiber is a limitation of its use in the deep or complex periimplantitis pockets. Then, lasers with more flexible fibers able to deliver a side firing irradiation may facilitate the access to the totality of the pocket and the efficient cleaning under the implant threads. The Diode and Nd:YAG (Neodymium-doped Yttrium Aluminum Garnet) lasers can be connected to very flexible fibers. 

Furthermore, the effect of the laser irradiation on the periodontium and the possible damage that the irradiation may make on the implant surfaces (i.e., morphological changes, defects…) must be taken into consideration. Actually, an increase in temperature during irradiation may lead clinically to dramatic effects such as the necrosis of surrounding tissue, damage of the implant and others [18]. The use of short pulse duration can seriously reduce the heat generated by the interaction of the laser beam with the implant surface and contaminants. In fact, the available Er:YAG laser apparatus for dental applications are not able to deliver short pulses in nanoseconds, they are limited to microseconds. Because of the increase of periimplantitis prevalence and the clinical necessity to have an efficient procedure for the treatment of peri-implantitis, the finding of a new apparatus able to deliver a very short pulses in nanoseconds allowing implant cleaning, with harmlessness, becomes important. Hence, the Q-switch Nd:YAG (Neodymium-doped Yttrium Aluminum Garnet) laser is able to deliver very short pulses in nanoseconds. 

In this in-vitro study, the possible benefit of using shorter laser pulses, (nanoseconds for the treatment of periimplantitis on a theoretical modeling basis) was investigated. The parameters were chosen according to the results of a theoretical modeling calculation of the Q-switch Nd:YAG laser interaction with the implant surface. The scanning electron microscopy (SEM) was used to analyze the effects produced by the laser beam on the implant surfaces and to detect any possible surface damage. Energy-dispersive X-ray (EDX) spectroscopy was used for the quantitative assessment of chemical elements present after irradiation. Additionally, the raise in temperature at implant structure, during the irradiation procedure, was measured in order to evaluate any possible thermal effect of our suggested protocol on the periodontal tissue surrounding the implant. 

Hence, the purpose of our study is to evaluate the cleaning of contaminated dental implant surfaces by means of the Q-switch Nd:YAG laser and the increase in temperature at lased implant surfaces during the cleaning process. The null hypothesis is that there is no significant difference between the cleaning of lased and un-lased implant surfaces. 

## 2. Materials and Methods 

### 2.1. Experimental and Control Group

Seventy-eight dental implants (titanium grade 4) in total (Euroteknika, Sallanches, France) were included in this study. Thirty-six sterile implants were considered as the control group (Groups S). The sterile implants of the Group S were kept in their own sterile packages until SEM and EDX examinations. Forty-two contaminated dental implants were collected from clinical failed cases with peri-implantitis. Only thirty-six contaminated implants were partially irradiated on their surfaces. The lased surfaces were considered as the group of the contaminated surfaces that were lased (Group C + L) and the non-lased surfaces of the contaminated implants were considered as the contaminated but unlased group (Group C) and was used as the second control group. The other six contaminated implants were used for the temperature increase measurements (Table 1).

The contaminated implants were collected from several dental clinics and from failed cases with PI (bone resorption, loss of osteointegration, etc.). Their removal was not, in any case, related to our study. Before experimentation and for the purpose of standardization, all contaminated implants were preserved, from collection until the experimentation, in sterile saline liquid of 0.9% NaCl and at a temperature of 37 °C to mimic the in vivo conditions in a constant temperature incubator (Heratherm IMH180SS, Thermo Fisher, Waltham, MA, USA). The solution was changed every 24 h until experimentations. 

No approval from the ethical committee of our university is necessary for this kind of study because the removal of the failed implants was not related in any way to our study.

### 2.2. Irradiation and Treatment Protocol

A Q-switch Nd:YAG laser 1064 nm wavelength (Q-smart 850, Lumibird, Lannion city, France) was used. A powermetter (Ophir, Ophir Spiricon Europe GmbH, Darmstadt, Germany) allowed the evaluation of the irradiation conditions to be effectively delivered on the target. Effective energy density per pulse of 0.597 J/cm^2^ was used, pick powers density of 56 mW/cm^2^, 270 mW per pulse with a spot diameter of 2.4 mm and with repetition rate of 10 Hz for pulse duration of 6 ns. Irradiation was performed in a non-contact mode at 0.5 mm distance from implant surfaces. The energy was chosen based on the theoretical modeling of the laser fluency at the level of damage threshold of the titanium surface (see below). 

The thirty-six contaminated implants were irradiated on the partial area of their surfaces during a total time of two seconds corresponding to 20 laser shots (10 Hz, 2 s). After irradiations, each fixture was stocked in a sterile box for analysis.

### 2.3. Theoretical Modelling and Calculation of the Laser Fluency

The interaction of laser pulses with metallic surfaces has been studied extensively in relation to the photodesorption process [19] and the damage threshold [20]. Hereafter, we apply these well-established theories to the laser decontamination of a titanium implant surface. The reflectivity (*R*) of the titanium surface is wavelength-dependent and is deduced from its complex refractive index (*n + i k*) using the Fresnel equation:

(1) $$R=\frac{{\left(1-n\right)}^{2}+k^{2}}{{\left(1+n\right)}^{2}+k^{2}}$$

As summarized in Table 2, titanium reflectivity at 0.532 µm laser KTP (Potassium titanyl phosphate) laser is as low as 52% and increases to 90% at 10 µm (CO_2_ laser), as expected from the improved screening of low-frequency electric fields by the metal electrons.

When the incidence angle departs from normal, the reflectivity becomes polarization-dependent, and the ‘averaged unpolarised’ reflectivity increases to reach 100% at grazing incidence. Upon reflection, the light is absorbed in a thin region underneath the metal surface corresponding to a penetration depth 1α (with α being the absorption coefficient) of a few tens of nanometers:

(2) 1α=λ4πk

**Figure 1:**
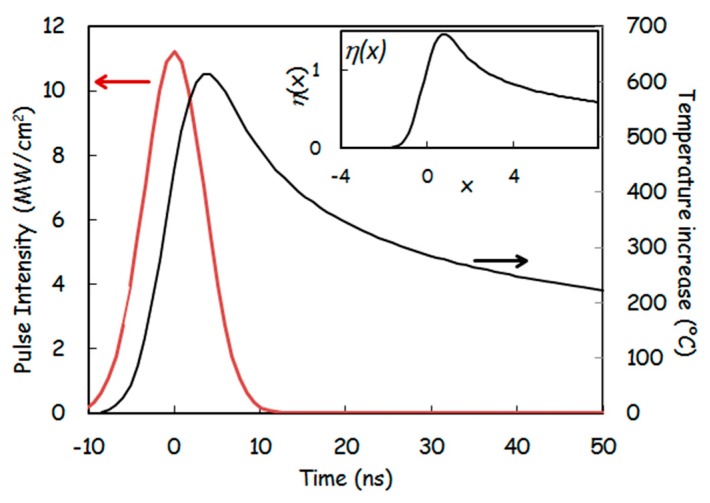
Increase in titanium surface temperature, ΔT(t), upon a single laser pulse radiation according to the parameters in Table 1. Inset: dimensionless function *η(x)* [21].

The rise in transient surface temperature can be determined by solving the heat diffusion equation:

(3) $$\frac{\partial T}{\partial t}=A\;\nabla^{2}T+\frac{S}{C\rho }$$

where, *T* is the local temperature, *A* is the thermal diffusion coefficient ($A=\frac{K}{C\rho }$), C is the heat capacity, ρ is the density, and *K* is the heat conductivity of titanium. *S* is the heat source corresponding to the local absorption of light. An analytical solution for the surface temperature rise (ΔT(t)) was derived by Bechtel et al. [21] for a laser pulse (I(t)) with a Gaussian temporal profile. The transient surface temperature (ΔT(t)) is given by:

(4) ΔT(t)=F (I−R)τ2πKρc η(tτ)

(5) $$I\left(t\right)=\frac{F}{\tau \sqrt{\pi }}\;\;e^{-{\left(\frac{t}{\tau }\right)}^{2}}$$

where, F and τ are pulse fluency and duration, respectively. $I_{m}=\frac{F}{\tau \sqrt{\pi }}$ is the peak intensity of the laser pulse. η(x) is a dimensionless function which can be derived from the parabolic cylinder functionsm [21] and is tabulated in the inset of Figure 1 which shows ΔT(t).

From Equation (3), we deduce that the maximum temperature rise at the surface ($\Delta T_{max})$ is inversely proportional to the square root of the laser pulse duration (τ):

(6) $$\Delta T_{max}=\frac{F\;\left(I-R\right)}{\sqrt{\tau }\sqrt{\sqrt{2}\pi K\rho c}}\;\eta_{max}=\frac{F\;\left(I-R\right)}{\sqrt{\tau }\sqrt{\sqrt{2}\pi K\rho c}}\;1.453$$

where, $\eta_{max}$ is the maximum value of *η(x).* This means that a Q-switch laser with a pulse duration of 6 ns can elevate the surface temperature with fluencies two orders of magnitude smaller than that of a laser working in relaxation mode with a pulse duration of 100 µs. The thickness of the metal layer, where most of the temperature gradient is localized, corresponds to the heat diffusion length (HDL). For the titanium substrate during the laser pulse, it is evaluated by quantity [22]:

(7) $$\mathrm{HDL}=\sqrt{A\;\tau }$$

The HDL decreases with shorter pulses. This means that if the damage threshold is reached when $\Delta T_{max}$ is higher than the titanium melting temperature, short pulses (<5 ns) will induce surface melting at a depth much smaller than the size of the implant surface microstructure. Therefore, despite surface melting, the shapes of the microstructure will be preserved. However, in the same condition, long laser pulses (>100 µs) will strongly modify the surface roughness (Table 3).

Finally, Equation (3) indicates that the transient temperature at the interface scales with the laser pulse duration, independently from the laser pulse energy. No cumulative effect of heat will occur if the laser pulse separation in time is two orders of magnitude larger than the pulse duration.

The thermal desorption process of a single ad-molecule obeys the Arrhenius equation relating the rate of a chemical reaction to the absolute temperature (*T*), a pre-exponential factor (*P*) and the activation energy of the reaction (*E_a_*):

(8) $$\frac{\partial C}{\partial t}=C\;P\;e^{\frac{-E_{a}}{K_{b}T}}$$

where, *C* is the adsorbed molecule coverage, $\frac{\partial C}{\partial t}$ is the desorption probability rate, and *K_b_* is the Boltzmann constant. When the complexity of the adsorbate increases and the interactions between ad-molecules must be taken into account, this rate equation must be adapted by defining, for example, the desorption order [23]. The kinetics of the thermal desorption of organic contaminants or biofilms from titanium are not documented. The process is complex and includes several intermediate states such as the thermal degradation of the contaminants. For the sake of simplicity, however, we will model the decontamination of the titanium surface using the simplest form of the Arrhenius equation given by Equation (8).

From Equations (6)–(8), we can observe different trade-offs. When shortening the laser pulses (τ), the pulse energy required to raise the surface temperature, as well as the HDL into the substrate, decreases with $\sqrt{\;\tau }$. However, the transient temperature duration decreases with τ which reduces the quantity of contaminant photo-desorption for the same value of $\Delta T_{max}$. This can be compensated by increasing $\Delta T_{max}$ since the desorption rate increases exponentially with that quantity. To evaluate these trade-offs, we will adopt desorption kinetics parameters which are typical for organic molecules adsorbed on metals and titanium oxide surfaces [24]. The pre-factor value is thus set to P = 10^14^ Hz. The adsorption energy is set to *E_a_* = 30 kcal/mol (1.3 eV/molecule) corresponding to a strongly chemisorbed organic molecule which would desorb near 300 °C during a slow thermal programmed desorption ramp of 1 °K/s. We will assume that the laser operator will adjust the laser power to prevent damage to the implant surface occurring when the maximum temperature rise, $\Delta T_{max}$, reaches the titanium melting temperature ($T_{melting\;}$ = 1668 °C). Since $\Delta T_{damage}=\;T_{melting}-\;T_{room}$ (with $T_{room}$ = 25 °C), we will use, as setting parameter, the ratio:

(9) $$S_{a}=\frac{\Delta T_{damage}}{\Delta T_{max}}$$

This safety parameter (*S_a_*) should be set at about 0.5–0.8 to preserve the implant surface topography. For the simulation, we further assume laser beam reflection coefficient of 61%. Figure 4 shows the remaining coverage of the contaminant after one laser shot as a function of the safety parameter (*S_a_*) and for pulse durations of 0.5 ns, 6 ns, 100 µs and 100 ms.

**Figure 2:**
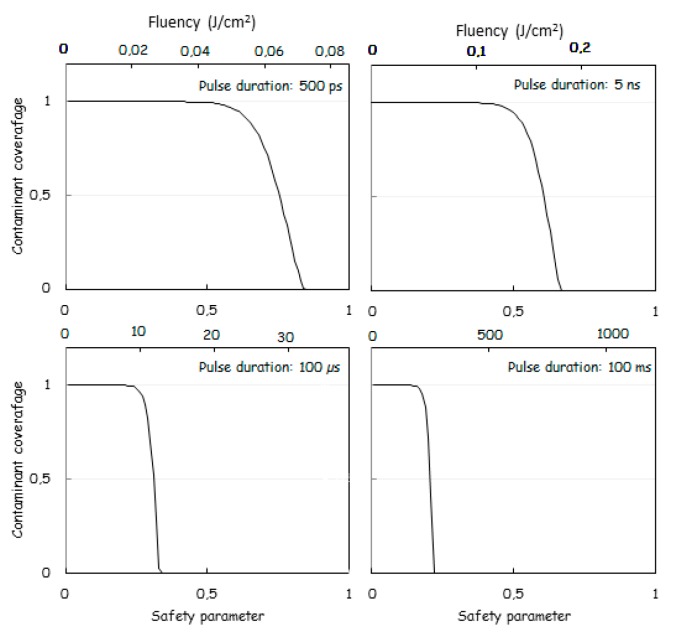
Remaining coverage of the contaminant after one laser shot as a function of the safety parameter (*S_a_*) and of the laser pulse duration.

Figure 2 shows the trade-off between the safety parameter and the pulse duration in order to achieve significant decontaminant desorption yield. Note that the safety parameter does not take into account the HDL which is much shorter in the case of a shorter pulse. This is in favor of short pulse durations, and this partly compensates, concerning the practical aspect of the safety, for the necessity to use a higher value of *S_a_* when using shorter pulses. Table 4 summarizes the laser specifications required to achieve proper decontamination as a function of pulse duration. We assume that the operator selects a fluency to achieve 90% decontamination per shot. Therefore, about 10 shots are applied to ensure a decontamination level of 99.99999999%. The treatment of a complete implant, the surface of which is about one cm^2^, requires about 2000 shots. Since the total optical energy impinging the implant, the implant volume (~5 × 10^−7^ m^3^), the titanium heat capacity and the reflectivity (61%) are known, the implant temperature rise (ITR) can be evaluated according to:

(10) ITR=F Sp 2000 (1−R)5 10−7m3 ρ c=3.2 °C(Jcm2) F

This modelling enables the prediction that a shorter pulse will improve the decontamination procedure. Very short pulses in the range of 500 ps appear advantageous. Further shortening of the laser pulse duration will not improve the decontamination process. Indeed, as observed for the measurement of metal surface damage thresholds [25], the limited transfer rate of energy between the electronic and atomic vibration degrees of freedom in metals means that absorbed optical energy is thermalized in the substrate on a time scale of the order of 0.5 ns. Therefore, shorter laser pulses will not entail a shortening of the transient surface temperature.

The present modelling is certainly oversimplified. Indeed, it does not take into account either the absorption of the contaminant or the roughness degree of implant surfaces which may increase the reflectivity of the beam resulting in a reduction its efficiency. For those reasons, in our study, we decided to use a slightly higher energy density of 0.597 J/cm^2^ which is slightly higher than the theoretical one.

### 2.4. Scanning Electron Microscope (SEM) Measurements of the Three Groups 

A scanning electron microscope (SEM) (JEOL 7600F, Jeol, Akishima, Japan) was used under 250× magnification, acceleration voltage of 15.0 kilovolt and working distance of 28 mm. This is to analyze the effect of the laser beam on the three areas of implant surfaces and to detect any eventual surface damage or alteration of the titanium. Samples from the three groups (S, C and C + L) were analyzed directly without any previous treatment or preparation and without being covered by any metallization process.

### 2.5. Energy Dispersive X-ray (EDX) Spectroscopy Measurements of the Three Groups

Energy dispersive X-ray spectroscopy (EDX, Oxford, UK) was used for the quantitative analysis of the chemical elements on the surfaces of all implant samples. The carbon (C), oxygen (O), calcium (Ca), Phosphorus (P), sodium (Na), Silicon (Si) derived from the implant surface were assessed for all concerned implant surfaces in all groups. The presence of the chemical element (mass %) on the surface of all samples was noted. Twelve points were randomly analyzed on each implant surface area of all groups. All measurements and observations were collected and noted. The percentage of the carbon atoms on the implant surface has been given a special attention due to its significance in the presence of germs, biofilm and other soiling or stains.

### 2.6. Temperature Increase Measurements

Six additional contaminated implants were used only to assess temperature increase during the protocol suggested. In this study, the measurements of temperature increase during irradiation following exactly previous protocols that were used for the same purposes [26,27,28]. To ensure optimal contact and maximal thermal conduction between the sensor tip of the thermocouple probe and the implant surface, a thermoconductor paste (warme Leitpaste WPN 10, Austerlitz Electronic, Nurnberg, Germany) was spread on a small area of two mm^2^ of implant surface and at one mm far from the irradiation area. The thermal conductivity of the paste was 0.4 cal s^−1^ m^–1^ K^−1^, and it is comparable to the thermal conductivity of soft tissues. (0.2 – 0.5 cal s^−1^ m^−1^ K^−1^ depending on hydration) [26,27,28].

**Figure 3:**
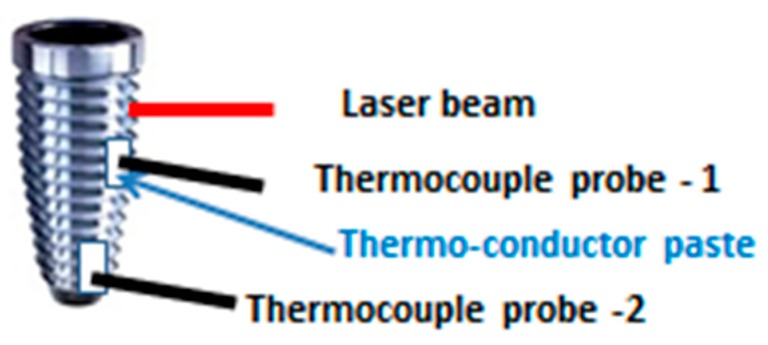
Setup for the assessment of the temperature increase measurements on a contaminated dental implant. The thermocouple probes placed in close contact with the apical region and at 1 mm from the irradiation area of implant surface.

A type K thermocouple was used (Model TM-946, 4 channels, Lutron, Taipei, Taiwan), with an accuracy of 0.01 °C. One thermocouple probe was placed in close contact with the implant surface at 1 mm far from the irradiation area. A second probe was placed at the implant apex (Figure 3).

Laser irradiation started when the temperature at the implant surface was constant and equal to the room temperature. Temperatures were recorded every second during irradiation and during a period of 180 s after irradiation. Five measurements were made on the surface of the implant for each irradiation parameter. The considered temperatures (*Δt*) were calculated as the difference between recorded temperatures at initial temperature (*T_I_*) and highest recorded temperatures (*T_H_*): *Δt* = *T_H_* − *Ti*.

### 2.7. Statistical Analysis

In order to analyze, the measurements obtained from the EDX statistical analyses were performed using GraphPad Prism program (GraphPad 6 Software, Inc, San Diego, CA, USA). Means and standard deviations (SD) of carbon mass (% mass) on implant surfaces were reported. One-way ANOVA followed by Newman-Keuls posttest for pairwise comparisons was used. A *p*-value of less than 0.05 was considered statistically significant. 

For the measurements of temperature increase during irradiation, the mean temperatures (*Δt*) and the standard deviation for each irradiation condition were calculated. Normality tests were carried out using the Kolmogorov and Smirnov test.

A biostatistician that was completely blind to the study conducted all the statistical analysis independently. 

## 3. Results

### 3.1. Scanning Electron Microscope (SEM) Analysis 

**Figure 4:**
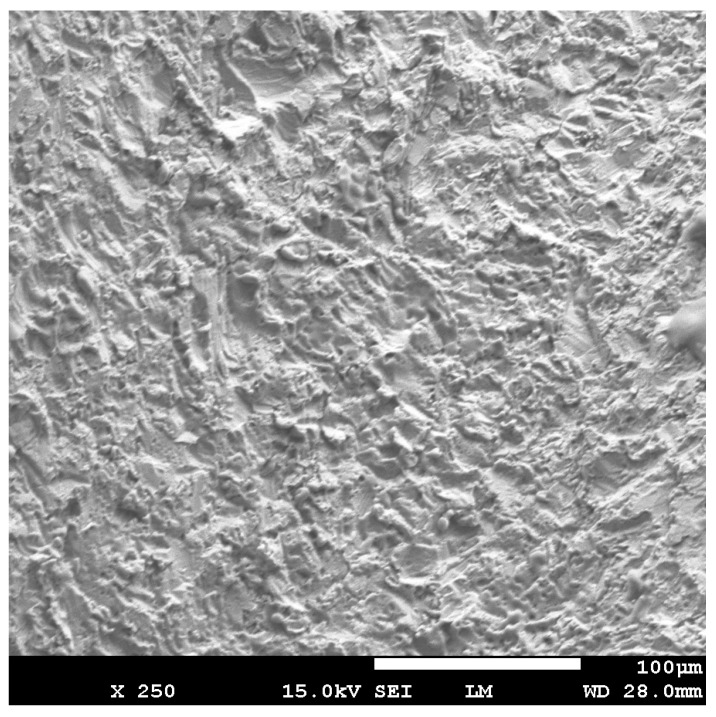
Scanning Electron Microscope (SEM) view of the lased area of contaminated implant (group C + L). The surface showed a clean aspect without alteration, melting areas or cracks.

**Figure 5:**
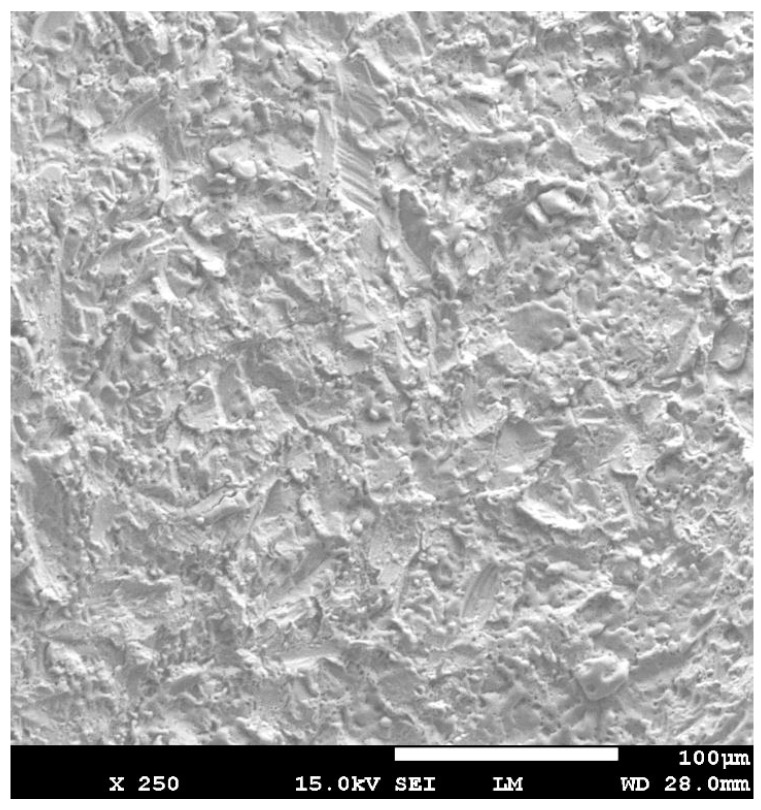
SEM view of the group S showing the relief and the roughness of the surfaces of a sterile implant.

**Figure 6:**
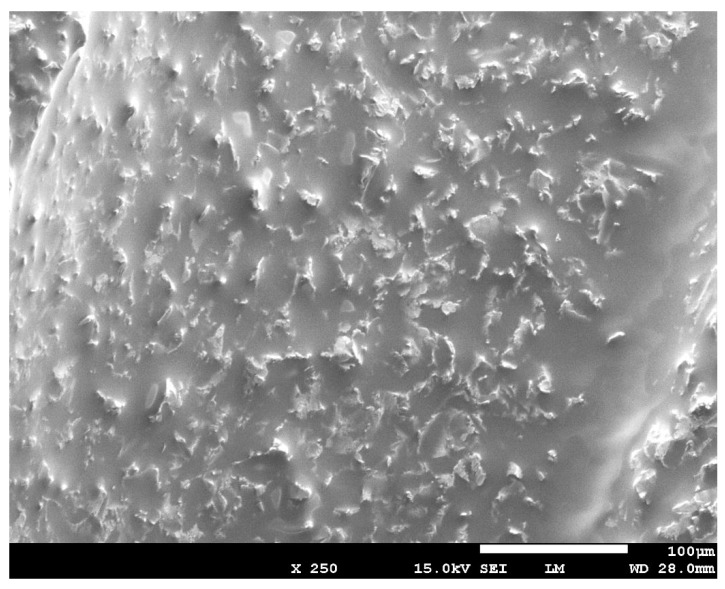
SEM view of the unlased but contaminated implant surface (Group C) showed the contaminants totally covering the roughness and the relief of the implant surface.

The irradiated surface of the contaminated implants (Group C + L) showed a clean aspect without any alteration of the implant surfaces (Figure 4). The aspect of the surface of the lased contaminated implant (group C + L) was similar to the surface of the sterile implant (Group S) (Figure 5). The surface roughness was not altered. The use of Q-switch Nd-YAG laser for implant cleaning did not show any cracks or melted areas on implant surfaces in all samples (Figure 8). The SEM view of the unlased but contaminated implant surface (Group C) showed the contaminants totally covering the roughness and the relief of the implant surface (Figure 6).

### 3.2. EDX Quantitative Measurements

**Figure 7:**
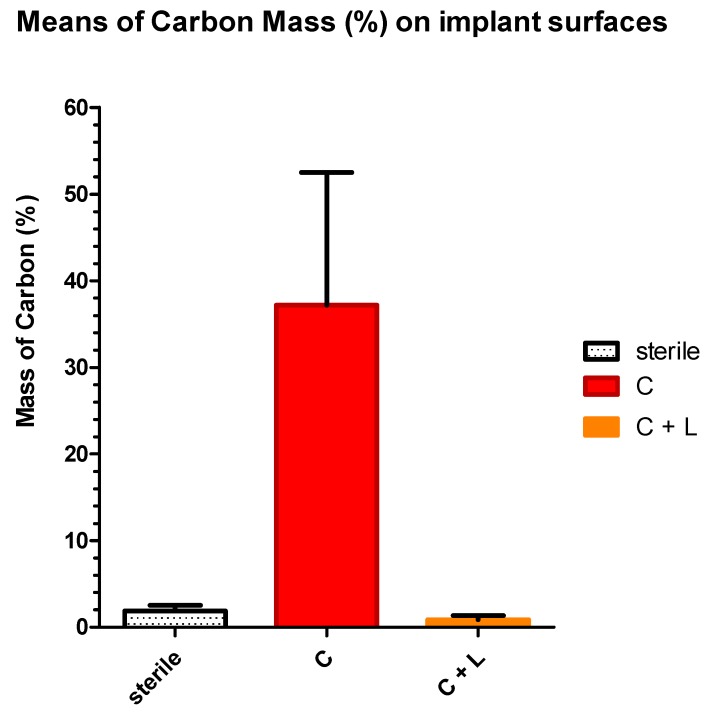
Percentage of mean of carbon mass (percentage) on implant surfaces in the S, C and L groups.

**Figure 8:**
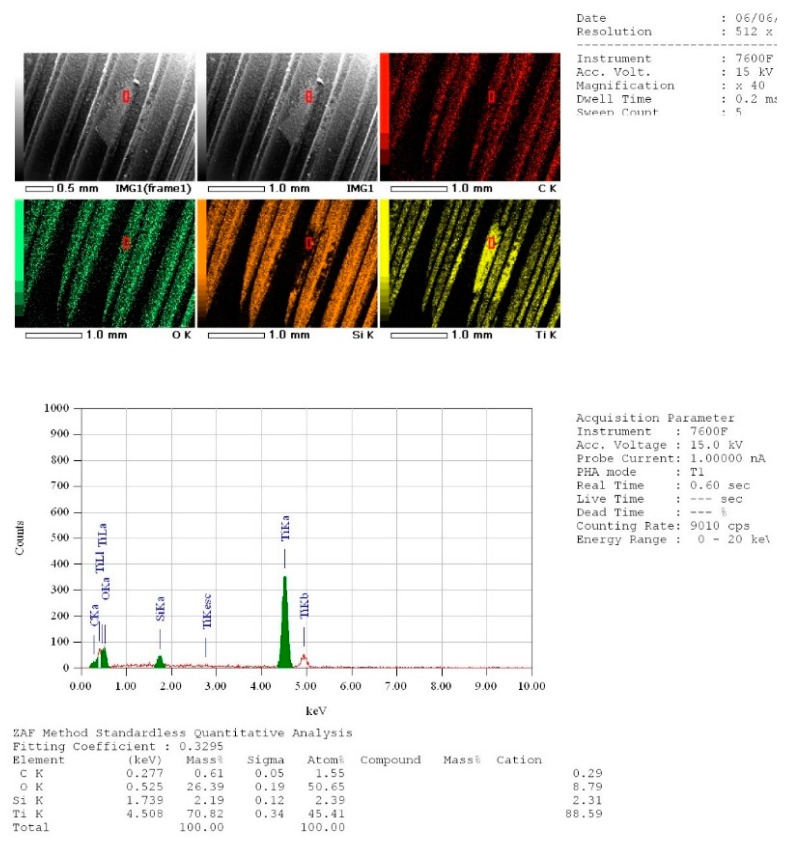
An example of an Energy Dispersive X-ray (EDX) measurement showing the value of Carbone (mass percentage) on lased surface of the group C + L.

The means and standard deviations of the carbon mass (percentage) were respectively 1.851 ± 0.672, 41.43 ± 10.41 and 0.975 ± 1.007 for sterile implants surfaces (Group S), contaminated implants surfaces (Group C) and for contaminated and lased implant surfaces (Group C + L) (Table 5, Figure 7). The values of samples in all groups have passed the normality test (KS test). The mean percentage mass of carbon measured on the implant’s surfaces showed a high significant difference (Newman–Keuls; *p* < 0.0001) between samples from group S (sterile) and group C (contaminated implants). The statistical difference was also highly significant (Newman–Keuls; *p* < 0.0001) between samples from Group C and those from Group L while the difference between group S and the group L was not statistically significant (Table 5). Since that, the presence of Carbone is an indication of contamination, the contaminated and lased implants showed surfaces that are significantly clean and similar to sterile implant surfaces (Figure 8). The null hypothesis was rejected.

### 3.3. Temperature Increase Measurements (°C)

The means and standards deviations of recorded temperature increase at implant surfaces during laser irradiation were respectively the following. 0.29 ± 0.049 °C, 0.17 ± 0.048 °C, 0.98 ± 0.040 °C and 0.56 ± 0.081 °C for 20 pulses (2 s) at 1 mm from the lased area, 20 pulses (2 sec) at the implant apex, 40 pulses (4 s) at 1 mm from the lased area and for 40 pulses (4 s) at implant apex (Table 6). All records were below the safety level of 10 °C for periodontal tissue injury. The data passed the normality test (KS) with *p* > 0.05. The one ANOVA test coupled to the post hoc Newman–Keuls Multiple Comparison Test showed that the means comparison in all group were significantly different with *p* > 0.001. 

## 4. Discussion

The biofilm theory is by far the most accepted theory to trigger most of peri-implantitis. The progression of PI is influenced by an inflammatory reaction caused by the oral biofilm even when surgical or loading factors are implicated [29]. Along the many risk factors, smoking is considered the main risk factor that may influence the appearance of peri-implantitis [30]. Mechanical elimination and decontamination of the titanium surface can be considered the most effective modality in the management of peri-implantits. The elimination of the implant surface contaminants will allow a reduction of inflammation and therefore a possible decrease in periodontal pocket depth and progressive bone regeneration [5]. 

Lasers were introduced in the management of periodontal and peri-implantitis diseases due to their following characteristics: reduction of damaged or inflamed tissues by photonic curettage of granulation tissues, the antimicrobial effects on biofilms in conjunction with an exogenous dye: application of antimicrobial photodynamic therapy/photoactivated disinfection (aPDT/PAD) and the photobiomodulation effect that promotes wound healing and tissue regeneration [31]. The absorption of the laser’s energy produces thermal changes such as protein denaturation (50 °C), protein coagulation (60 °C), vaporization and ablation (100 °C) and tissue carbonization if the temperature is greater than (200 °C) [32]. The heat generated by laser beam may damage the implant surfaces. For that reason, in our study, we made a theoretical modeling allowing us to use an optimal and low energy density with short pulses in nanoseconds in order to reduce significantly the heat generation and to stay below the threshold level of the implant titanium damage. This study focused primordially on the ability of the Q-switch Nd:YAG laser to clean the dental implant surfaces. Analysis of the surface composition of the dental implants after irradiation, SEM analysis and the increase in temperature during irradiation were carried out in order to assess the efficiency of our suggested protocol.

Thermal-Photo-Desorption is defined as a thermal effect generated by a light, resulting in a desorption (removal) of a substance from a metallic surface. The photo-desorption of organic molecules from a metal surface has been the subject of several studies [33] with the objective of discerning the resonant and thermal character of the photo-desorption process. Even if a metallic surface is irradiated in the UV range, the breaking of the adsorbate–substrate bonds requires the absorption of multiple photons [34]. This condition is even more severe in the infrared spectrum, where the photon energy is much less than the molecule adsorption energy. In all cases, substrate absorption is the dominant mechanism of energy uptake from the laser beam. Therefore, in the infrared range, thermal processes dominate the laser-induced photodesorption (LIPD) process. In physics, it is well established that the property in the infrared is relatively wavelength independent, but temperature transience metal absorption and contaminant desorption are strongly pulse duration dependent. Biological tissue absorption can be in the order of = 1 cm^−1^ near 1.064 µm, and 100 cm^−1^ near 2.94 µm [35]. Therefore, it cannot be concluded that 100 µm-thick biofilms can show significant absorption in the order of 1% or 65%, near 1064 and 2940 nm, respectively. The thermal diffusion coefficient of biological tissues being of the order of A ~1.4 × 10^−3^ cm^2^/s (Boulnois, 1986), thermal equilibrium through the biofilm is not guaranteed since the diffusion length for 5 ns is $\sqrt{A\;\tau }$ ~26 nm, which can be significantly less than the biofilm thickness. Also, as mentioned earlier, the desorption kinetics of implant contaminants are unknown. However, the complexity of these phenomena will not fundamentally change the trade-offs between the transient surface temperature duration and amplitude and the exponential behavior of the desorption process, as modelled in the present calculations. As a conclusion, shorter pulse duration is advantageous for implant surface decontamination. For this reason and in order to select the optimal energy density that may lead to optimal value of energy density with minimal heat generation (below the threshold level of the implant titanium damage) but able to accomplish an efficient photo-desorption of the contaminants, a prior theoretical modeling and calculation of the laser fluency has been made. The theoretical modeling and calculation showed that shorter pulses in the range of 5 ns to 0.5 ns are preferable. In addition, the shorter pulses in the range of 5 ns to 0.5 ns lead to a reduction of up to two orders of magnitude of the required laser output power and consequently, a reduction of the implant temperature rise during the decontamination process.

Concerning the findings of our in vitro study, qualitative assessments of the chemical composition on the lased surfaces of the contaminated implants showed total absence of Carbone component, which indicates that the laser irradiation was able to photo-desorb and to efficiently clean the surfaces. Our results confirmed that the Nd:YAG laser when used with our specific protocol, and parameters lead to significant cleaning, by photo-desorption, of the dental implants, which was confirmed by the fact that a non-significant mean value difference was found between the surface composition of the sterile implants and the lased contaminated implant. 

On the other hand, the safety of the procedure on the periodontal tissue was also studied. The mean increase in temperature during the irradiation was less or equal to 1 °C. The increase in temperature during the irradiation indicated that the delivered energy was not high enough to produce a morphological damage on the implant surfaces. In fact, all recorded measurements of temperature increase were below the safety level of 10 °C for periodontal tissue injury. Hence, these findings confirm the safety of the protocol on the periodontal tissue.

The potential damage of the implant surface due to the irradiation was carried out in this study. Qualitative analysis using scanning electron microscopy did not detect any eventual surface damage or alteration of the titanium in the contaminated and lased surfaces. No cracks or melted areas on the implant surfaces were detected, and the roughness of the surface was not affected.

The use of lasers and its effect on dental implant surface has been extensively studied in literature. According a literature review conducted by Subramani et al., the lasers and photodynamic therapy have showed benefice [36]. In contrast, a systematic review and meta-analysis, failed to show an additional advantage of the lasers in periodontal pocket reduction when compared to conventional implant surface decontamination methods [37]. 

The use of the CO_2_ laser did not show any alterations such as crater-like, melting or lava-like layers when used in specific parameters, suggested by Shibli et al. [38]. Mouhyi et al., showing that a combination of citric acid, hydrogen peroxide, and CO_2_ laser irradiation is effective for cleaning and reestablishment of the atomic composition and oxide structure of contaminated titanium surfaces [39]. In accordance with the previous studies, several studies have also shown positive outcomes and promising results when the CO_2_ laser was used in order to treat PI [40,41,42,43]. 

The use of the Erbium lasers Er:YAG and Er,Cr:YSGG (Erbium, chromium-doped yttrium, scandium, gallium and garnet) had also been widely described. According to Ecran et al., and within their parameters, the Er,Cr:YSGG laser resulted in alterations such as melting, flattening and, in some, cases a cracking was notable. Therefore, Ecran et al., concluded that optimization of the parameters should be made before any irradiation [38]. In contrast, Nevins M. et al., demonstrated in a preclinical canine study, that the use of the Er:YAG laser showed an arrest of the progression of periimplantitis and an evidence of a formation of new bone-to-implant contact [44]. An in-vivo study conducted by Schwarz et al., revealed that an effective removal of subgingival calculus with residual amounts of debris without any thermal damage was made with the Er:YAG laser [45]. 

The Q-switch Nd:YAG laser was able to promote a total reduction of contamination on contaminated implant surfaces with Enterococcus faecalis and Porphyromonas gingivalis according to an in-vitro study by Goncalves et al. [46]. The Nd:YAG group showed higher decontamination when compared to a group treated only with sand-blasted TiO and acid-etched surfaces. In addition, Vassalli et al., demonstrated that when the Nd:YAG laser was used with lower energy of 30 mJ, there was no morphological modification of the lased implants; therefore, the safety of the use of Nd:YAG laser within their specifications was confirmed [47]. In opposition, Block et al. showed that the Nd:YAG laser did not decontaminate infected implants with Bacillus subtilis [48]. As already mentioned, peri-implantitis can be induced by many factors related to oral hygiene, smoking habits, genetics, systematic disease, iatrogenic causes and others. In addition to these factors, periimplantitis is also influenced by the macro and microstructure of the implant. In fact, the study on dogs of Fickl et al. concluded that “Nobel replace tapered” implants are associated with pronounced tissue loss [49]. 

Further studies are necessary to confirm the effectiveness of Q-switch Nd:YAG laser to remove biofilms and germs from the contaminated implant surfaces in order to convert the non-biocompatible contaminated surface to a clean and biocompatible surface, allowing osseo-integration and bone regeneration.

## 5. Conclusions

The findings of this study suggest that Q-switch Nd:YAG laser irradiation with the short pulse duration in nanoseconds is able to significantly clean the contaminated implant surfaces. The irradiation condition used in this study can be considered safe for periodontal tissue generating an increase of temperature at the implant surface around 1 °C. As a clinical implication, the use of the Q-switch Nd:YAG laser can be considered as a promising technique for the treatment of peri-implantitis.

## Figures and Tables

**Table 1:** Experimental and Control Group.

Groups	Number of Samples	Description
Group S	36	Sterile implants with untreated surfaces used as a control group.
Group C and Group C + L	36	Partial surfaces of the contaminated implants were kept unlased (second control group)
		Partial surfaces of contaminated implant of group C were treated with Q-switch Nd:YAG laser.
Group T	6	Group used for the temperature rise measurements

**Table 2:** Titanium optical properties.

Wavelength (λ)	*n*	*k*	Reflectivity (Equation (1))
0.532 µm (KTP laser)	2.2080	2.8980	53%
1.064 µm (Nd:YAG laser)	3.3731	3.9777	61%
2.94 µm (Er:YAG laser)	3.6872	5.2688	70%
10 µm (CO_2_ laser)	9.6000	18.600	92%

**Table 3:** Relation between laser pulse duration, fluency and heat diffusion length (HDL).

Laser pulse Duration	Pulse Fluency for Obtaining $\Delta T_{max}\;\;=\;1000\;{}^{\circ}C$	Heat Diffusion Length $HDL=\sqrt{A\;\tau }$
100 µs	22 J/cm^2^	25 µm
6 ns	0.16 J/cm^2^	196 nm
500 ps	0.05 J/cm^2^	62 nm

**Table 4:** Laser parameters for decontaminating implants. The laser fluency is set to achieve 90% decontamination per laser shot on a spot diameter of 800 µm. The implant is decontaminated by 20 shots on each spot. Complete surface decontamination of 1 cm^2^ is achieved by decontaminating 200 spots.

Laser Pulse Duration	Safety Parameter (*S_a_*)	Heat Diffusion Length (HDL)	Fluency (*F*)	Pulse Energy	Implant Temperature Rise (ITR) for 2000 Shots
100 ms	0.2	800 µm	145 J/cm^2^	0.73 J	464 °C
100 µs	0.32	25 µm	12 J/cm^2^	60 mJ	38 °C
6 ns	0.14	196 nm	0.18 J/cm^2^	850 µJ	0.54 °C
600 ps	0.81	62 nm	0.07 J/cm^2^	350 µJ	0.22 °C

**Table 5:** Mean values (standard deviation (SD)) of Carbon Mass (%) analysis according to groups. Lowercase superscript letters indicate statistically significant differences (Newman–Keuls; *p* < 0.0001) between groups.

Groups	Sterile (Group S)	Contaminated (Group C)	Contaminated and Lased (Group C +L)
Number of values	36	36	36
Mean (SD)	1.851 (0.672) ^a^	41.43 (10.41) ^b^	0.975 (1.007) ^a^
Std. Error	0.07609	1.480	0.1808

**Table 6:** Means and standard deviations are shown for each irradiation condition. SD: Standard deviation. Lowercase superscript letters (a, b, c and d) indicate statistically significant differences (Newman–Keuls; *p* < 0.0001) between groups.

Irradiation Conditions	20 Pulses, 10 HZ (Impact)	20 Pulses, 10 Hz (Apex)	40 Pulses, 10 Hz (Impact)	40 Pulses, 10 Hz (Apex)
Number of values	36	36	36	36
Mean (SD)	0.29 (0.0493) ^a^	0.17 (0.0480) ^b^	0.98 (0.040) ^c^	0.56 (0.081) ^d^
Std. Error	0.01369	0.01332	0.01667	0.03333
